# Disparities in HIV Clinical Stages Progression of Patients at Outpatient Clinics in Democratic Republic of Congo

**DOI:** 10.3390/ijerph18105341

**Published:** 2021-05-17

**Authors:** Raimi Ewetola, Gulzar H. Shah, Lievain Maluantesa, Gina Etheredge, Kristie Waterfield, Astrid Mulenga, Apolinaire Kilundu

**Affiliations:** 1 Division of Global HIV and Tuberculosis, CDC, Atlanta, GA 30329, USA; hcx6@cdc.gov; 2 Jiann-Ping Hsu College of Public Health, Georgia Southern University, Statesboro, GA 30460, USA; kwaterfield@georgiasouthern.edu; 3 FHI 360, Kinshasa 1015, Democratic Republic of the Congo; LMaluantesa@fhi360.org (L.M.); AMulenda@fhi360.org (A.M.); 4 FHI 360, Washington, DC 20009, USA; GEtheredge@fhi360.org; 5 National AIDS Control Program (PNLS), HIV Program, Ministry of Health, Kinshasa 316, Democratic Republic of the Congo; apogkilundu@gmail.com

**Keywords:** HIV clinical stages, TB/HIV co-infection, antiretroviral treatment, multinomial logistic regression, Democratic Republic of Congo

## Abstract

Context: In this era of patient-centered care, it is increasingly important for HIV/AIDS care and treatment programs to customize their services according to patients’ clinical stage progression and other risk assessments. To enable such customization of HIV care and treatment delivery, the research evidence explaining factors associated with patients’ clinical stages is needed. Objectives: The primary objective of this study was to produce such scientific evidence by analyzing the most recent data for patients at outpatient clinics in the provinces of Kinshasa and Haut-Katanga and to examine the patient characteristics associated with WHO stages of disease progression. Methods: Using a quantitative retrospective cohort study design, we analyzed data from 49,460 people living with HIV (PLHIV) on antiretroviral therapy (ART) from 241 HIV/AIDS clinics located in Haut-Katanga and Kinshasa provinces of the Democratic Republic of Congo. We performed Chi-square and multinomial logistic regression analyses. Results: A small proportion (i.e., 4.4%) of PLHIV were at WHO’s clinical progression stage 4, whereas 30.7% were at clinical stage 3, another 22.9% at stage 2, and the remaining 41.9% were at stage 1, the least severe stage. After controlling for other demographic and clinical factors included in the model, the likelihood of being at stage 1 rather than stage 3 or 4 was significantly higher (at *p* ≤ 0.05) for patients with no tuberculosis (TB) than those with TB co-infection (adjusted odds ratio or AOR, 5.73; confidence interval or CI, 4.98–6.59). The odds of being at stage 1 were significantly higher for female patients (AOR, 1.35; CI, 1.29–1.42), and those with the shorter duration on ART (vs. greater than 40.37 months). Patents in rural health zones (AOR, 0.32) and semi-rural health zones (AOR, 0.79) were less likely to be at stage 1, compared to patients in urban health zones. Conclusions: Our study showed that TB co-infection raised the risk for PLHIV to be at the severe stages of clinical progression of HIV. Such variation supports the thesis that customized HIV management approaches and clinical regimens may be imperative for this high-risk population. We also found significant variation in HIV clinical progression stages by geographic location and demographic characteristics. Such variation points to the need for more targeted efforts to address the disparities, as the programs attempt to improve the effectiveness of HIV care and treatment. The intersectionality of vulnerabilities from HIV, TB, and COVID-19-related hardships has elevated the need for customized care and treatment even more in the COVID-19 era.

## 1. Introduction

Since the mid-1980s, the epidemic of human immunodeficiency virus (HIV) infection and acquired immunodeficiency syndrome (AIDS) has been a significant global public health concern [1]. HIV is a highly variable disease that progresses through stages; however, the rate of progression between individuals can be categorized as rapid, typical or intermediate, and late or long-term non-progression [2,3,4,5]. In countries and areas that have the necessary resources, laboratory testing is used to monitor a patient’s disease progression, degree of immunosuppression, and eligibility for treatment. In many resource-limited settings, including most of the regions hardest hit by the HIV/AIDS epidemic, access to laboratory testing is limited at best [6]. The clinicians and health care workers in these areas must rely on clinical parameters when assessing a patient’s disease progression [7]. In 1985, the World Health Organization (WHO) defined the clinical case definitions of both HIV and AIDS; in 1990, they developed the HIV/AIDS clinical staging system to emphasize the use of clinical parameters to guide clinical decision-making for patient management; and in 1994, they redefined the clinical case definitions so that they could be used in resource-limited settings. The WHO clinical staging system categorizes patients into one of the tiered clinical stages: stage 1 (asymptomatic), stage 2 (mildly symptomatic), stage 3 (moderately symptomatic), and stage 4 (severely symptomatic or AIDS) [6]. For patients to move into a higher stage, the patient must exhibit at least one of the clinical conditions within the next higher stage’s criteria; once a patient is moved into a higher stage, they remain within that stage until they exhibit at least one clinical condition of a higher stage [8].

The rate at which a patient moves through the HIV/AIDS treatment cascade and progression through the clinical stages are affected by many different factors. In areas with access to laboratory resources, both disease and treatment progression focus on immunological (such as T-cell count and immune activation) and virological (such as HIV-RNA viral load and resistance mutations) factors, while also taking into account the factors that affect access to health care, age, gender, and other sociological disparities [9]. In resource-poor areas with limited or no access to laboratory resources, measurement of disease and treatment progression focuses on simple markers such as delayed-type hypersensitivity responses (DTH), total lymphocyte count (TLC), hemoglobin, and body mass index (BMI) [9,10]. In these areas, disease progression measurement also takes into account the factors of insufficient health care infrastructures, scarcity of providers to distribute care, limited training for providers, poverty, disease stigma, cultural and social barriers to testing and treatment, lack of health literacy, and shortage of medical equipment and medications [9,10]. Many of these socioeconomic and cultural factors also contribute to the disproportionate impact of HIV in these regions [7].

The Democratic Republic of Congo (DRC) is one of many areas in Sub-Saharan Africa faced with the challenges of limited access to care and treatment, medication stock-outs, and limited or no access to laboratory services for HIV patients [11]. DRC is also considered one of the 30 countries that account for 89% of all new HIV infections and thus has been labeled as a “Fast-Track” country by WHO and the Joint United Nations Program on HIV/AIDS (UNAIDS). Fast-Track countries experience an accelerated pace in which the resources for prevention and treatment will become available thus adverting the rate of new infections. These resources will be based on each country’s national response strategy and will include extensive human, financial, and institutional mobilization [11]. Being identified as a “Fast-Track” country, DRC is afforded the opportunity by UNAIDS and other global partners to strengthen their HIV response by accelerating their prevention and treatment services, adapting the delivery of their services to ensure maximum efficacy and efficiency, and increasing investment while reallocating resources for greatest effectiveness [11]. However, challenges faced by national and provincial health system structure and the HIV programs and individual health centers (such as personnel and equipment shortages, lack of financial resources, and limited availability of electricity and running water) as well as patient characteristics, such as age, gender, and access to the programs and centers, are major factors that are difficult to overcome and can disproportionately affect the rate of a patient’s disease progression and their response to the treatment, antiretroviral therapy (ART) [6,7,9]. Additionally, throughout the clinically latent (asymptomatic) period of the HIV infection, the virus continues to actively replicate, which affects the progression of the disease and leads to a patient becoming symptomatic, thus contributing to the inherent need that ensures that the lifestyle factors that affect disease progression are identified and monitored closely during this asymptomatic period [9].

In 2018, only 62% of persons in DRC living with HIV knew they were HIV-positive [12], which means that of the more than 35% of persons who are infected but do not know it, once they are diagnosed and seek the treatment they will more than likely be in the advanced stages of the disease (defined as WHO stage 3 or stage 4), potentially hindering their response to ART [13]. Due to the gaps between those who have HIV, being aware of their status, and effectively receiving treatment, many of the treatment programs use the treatment cascade model to identify weaknesses and variations between the stages. The goals of HIV programs are to have early initiation of ART, slow disease progression, and achieve viral suppression, which are all very important to maintaining a patient’s quality of life. The treatment cascade for people living with HIV is defined as the steps in which the patients must progress through starting with initial diagnosis to achieving viral suppression. Viral load suppression is the final objective for those programs utilizing the HIV treatment cascade and is used by the program staff to gauge the patient’s response to ART [13]. In countries like the DRC, viral progression and the treatment cascade model help identify the proportion of persons living with HIV engaged at each WHO clinical stage and assist policymakers by highlighting the weaknesses at each stage as well as determine unacceptable variations between different populations [14].

Understanding the factors that affect the population progression along the clinical stage continuum between the clinical stages for HIV patients at outpatient clinics within the DRC will assist the decision-making processes regarding the effectiveness of the prevention, treatment, service delivery, and resource allocation. Currently, there are gaps within the research that specifically address the impact of patient characteristics and behaviors that affect HIV disease progression, viral suppression, and overall program effectiveness among outpatient clinics in DRC. To this effect, the purpose of this study is to examine the patient characteristics associated with WHO’s clinical stage for PLHIV receiving ART at outpatient clinics within the provinces of Kinshasa and Haut-Katanga.

## 2. Materials and Methods

### 2.1. Data

This research used a retrospective cohort study design based on secondary data. The study setting included 241 HIV/AIDS clinics supported by the Centers for Disease Control and Prevention (CDC) through the President’s Emergency Plan for AIDS Relief (PEPFAR) in two provinces of the Democratic Republic of Congo, namely Haut-Katanga and Kinshasa. The study data were extracted in June 2019, which covered 23 health zones: 11 health zones in Haut-Katanga, namely Kafubu, Kasenga, Kilwa, Pweto, Kashobwe, Kipushi, Katuba, Kisanga, Kowe, Mumbunda, and Tshamilemba health zones; and 12 health zones in Kinshasa, namely Binza Ozone, Kimbanseke, Kingabwa, Mont Ngafula 1, Masina I, Kinshasa, Limete, Lingwala, Matete, Ngaba, Nsele, and Ndjili. The study participants were 49,460 people living with HIV (PLHIV) who were on ART. The clinics from which the data were obtained are owned by the DRC government, as well as private and faith-based organizations, and are supported by the implementing partners participating in the National HIV/AIDS Program (PNLS), and with the support of PEPFAR. The implementing partners use a clinical database to track HIV counseling, testing, and service delivery encounters using the electronic patient management system TIER.Net, developed by the University of Cape Town. Georgia Southern University’s Institutional Review Board approved the study under the project protocol number H l9260.

### 2.2. Dependent Variable—WHO Clinical Stage

The dependent variable, WHO clinical stage at the time of the last visit, originally included stages 1 through 4. Because the WHO clinical stage 4 category had a small proportion of patients (3.4%), for the multivariate logistic regression model, the final two stages were combined, resulting in categorization of stage 1 (asymptomatic), stage 2 (mildly symptomatic), and stages 3 or 4 (moderately or severely symptomatic, respectively). The last category was used as the reference category in our multinomial logistic regression analysis.

### 2.3. Independent Variables

The independent variables included TB status of patient, patient sex, age at the time of the first visit, province, rurality status, health zone, and duration on ART. TB status variable was coded as a dichotomous variable, namely TB present or TB not present. Sex of patient was a dichotomous variable, recorded as “Male” and “Female”. Age of patient (at the time of the first visit) originally captured as a continuous variable, was dichotomized as younger than 15 years, and 15 years or older, because the two groups have distinct challenges and according to WHO and UNAIDS, age was defined by these two groups for surveillance purposes. [1,12] age of the patient was computed based on two variables, first visit date and patient’s date of birth.

The variable province contained two options, Haut-Katanga and Kinshasa, based on the geographic location of the clinic. The variable rurality consisted of three categories based on the health zones in which clinics were located: rural, semi-rural, and urban. Rural health zones included Kafubu, Kasenga, Kilwa, Pweto, and Kashobwe. The semi-rural zones consisted of Kipushi, Mont Ngafula 1, and Nsele, whereas the urban zones included Binza Ozone, Katuba, Kimbanseke, Kingabwa, Kinshasa, Kisanga, Kowe, Limete, Lingwala, Masina I, Matete, Mumbunda, Ndjili, Ngaba, and Tshamilemba. The variable duration on ART (months) at the time of the last reported visit in the data was computed based on the original variables included in the dataset “ART start date” and “date of the last visit for ART.”

### 2.4. Analytical Methods

For contextual information about the clinical characteristics and demographics of study participants, descriptive statistics such as frequency distribution, percentages, and arithmetic means were computed for all independent and dependent variables. Bivariate associations between the categorical independent variables and the dichotomous dependent variable were assessed using the chi-square test. Multinomial logistic regression was performed to estimate the nature of association of the independent variable, TB status, and the categorical dependent variable, WHO clinical stage, after controlling for other variables such as patient age, sex, province, rurality of health zone, and duration on ART. The significance of associations was determined based on *p* ≤ 0.05. All analyses for this study were performed using IBM SPSS Statistics version 25.0 (IBM Corporation, Armonk, NY, USA) [15].

## 3. Results

Among the patients receiving ART at HIV/AIDS clinics of the National HIV/AIDS Program (PNLS), 4.4% were at stage 4, the severely symptomatic stage, and 30.7% at clinical stage 3, the moderately symptomatic stage, for a combined total of 35.2% at stage 3 or 4. Those at the mildly symptomatic stage or WHO stage 2 constituted 22.9% of all patients. The largest proportion, 41.9%, was at stage 1, considered the asymptomatic stage (Table 1). A small proportion of PLHIV, 3.6%, had TB as a comorbidity. More than 80% of the patients receiving ART were treated at HIV clinics that were located in an urban health zone. Other descriptive statistics are provided in Table 1.

Table 2 shows that a significant bivariate association existed (*p* ≤ 0.05) between the TB status of the patient and WHO clinical stage, as 34.0% of persons with no TB and 67.9% of those with TB as a comorbidity were at WHO clinical stage 3 or 4. Additionally, significant differences existed by the client’s sex, as a smaller proportion of females than males (33.7% vs. 38.4%) was at WHO clinical stages 3 or 4. There was a small statistically significant difference by age, with a minuscule difference in the percent of patients younger than 15 years at WHO clinical stages 3 or 4 than older patients (35.5% vs. 35.6%). A small percentage of patients in Haut-Katanga (27.1%) compared to those in Kinshasa (36.4%) were at the WHO stage 3 or 4. Significant differences in percent at WHO stage 3 or 4 also existed by the number of months that a client was on ART; less than 3.23 months (30.6%), 3.23 to 14.52 months (30.0%), 14.53 to 40.37 months (35.2%), and more than 40.37 months (47.0%). Some significant differences existed in the percent of patients at certain WHO stages by province, health zone, and rurality status of health zone.

Table 3 presents the results of the adjusted multinomial logistic regression model with adjusted odds ratios (AORs) for each patient characteristic, with the group of patients in WHO clinical stages 3 or 4 as the reference group. Characteristics significantly associated with being at WHO clinical stage 1 (vs. being at stage 3 or 4) at *p* ≤ 0.05 were patient’s TB status, sex, province, rurality status of the health zone, and duration of being on ART (Table 3). The age of the client at the time of the first visit had no significant association with the WHO clinical stage. To elaborate, not having TB as a comorbidity was associated with much higher odds of being at a less severe clinical stage (WHO stage 1) rather than being at a moderately or severely symptomatic stage (adjusted odds ratio or AOR, 5.73; confidence interval or CI, 4.98–6.59). Female patients had higher odds of being at stage 1 rather than stage 3 or 4 (AOR, 1.35; CI, 1.29–1.42), indicating a higher risk for male patients to be at stage 3 or 4—the moderately or severely symptomatic stage, respectively. Rurality status was associated with being at the higher severity stage. Compared to patients in the urban health zones, those in the rural health zones had lower odds of being at the asymptomatic stage, stage 1 (AOR, 0.32; CI 0.29–0.36); those in the semi-rural health zones also had lower odds of less severe clinical stage 1 (AOR, 0.79; CI, 0.74–0.85). While the shorter the duration on ART was associated with being at a lower severity stage, patients that were on ART less than 3.23 months (AOR, 2.47; CI, 2.31–2.64), 3.23 to 14.52 months (AOR, 2.60; CI, 2.43–2.77), and 14.53 to 40.37 months (AOR, 1.77; CI, 1.65–1.88) had higher odds for being at stage 1 than stage 3 or 4 when compared to patients that were on ART for more than 40.37 months.

Being at WHO clinical stage 2, the mildly symptomatic stage (vs. being at stage 3 or 4) was also significantly associated with the patient’s TB status, sex, age, province, rurality status of the health zone, and duration of being on ART. Not having TB as a comorbidity was associated with the higher odds of being at WHO stage 2 rather than stage 3 or 4 (AOR, 3.60; CI, 3.10–4.20).

Female patients had higher odds of being at stage 2 rather than stage 3 or 4 (AOR, 1.09; CI, 1.03–1.15), indicating a higher risk for male patients to be at the moderately or severely symptomatic stage rather than asymptomatic stage. Province was associated with being at a lower severity stage. When compared to patients in Kinshasa, those in the Haut-Katanga province had higher odds of being at stage 2 rather than stage 3 or 4 (AOR, 1.85; CI, 1.73–1.98). Patients in rural health zones had lower odds of being at the mildly symptomatic stage, stage 2 (AOR, 0.50; CI, 0.44–0.56) compared to those in urban health zones. Patients in semi-rural zones did not have significantly different odds of being at WHO stage 2 rather than stage 3 or 4. Finally, when compared to patients with a duration on ART of more than 40.37 months, patients with a duration on ART of less than 3.23 months (AOR, 1.74; CI, 1.61–1.87), 3.23 to 14.52 months (AOR, 1.54; CI, 1.43–1.66), and 14.53 to 40.37 months (AOR, 1.28; CI, 1.19–1.38) had higher odds for being at stage 2 than stage 3 or 4.

## 4. Discussion

This study examined whether TB as a comorbidity was associated with WHO’s hierarchical clinical stages of HIV progression. The study data spanned from January 2014 to June 2019, pertaining to 49,460 HIV patients who were on ART in 241 HIV/AIDS clinics participating in the National HIV/AIDS Program in two provinces of the Democratic Republic of Congo, Haut-Katanga and Kinshasa. The study provides important research evidence for improving HIV care provision in these and other clinics in DRC, given that HIV continues to be a public health threat in Sub-Saharan Africa. The burden is evident from the statistics that 6.1 million people living with HIV are in the west and central regions of Sub-Saharan Africa, of which only 48% know their HIV status [1].

A small proportion of the PLHIV in this study, 3.6%, presented with TB as comorbidity. However, an overwhelming majority (67%) of these TB co-infected PLHIV were in stages 3 or 4, the most severe stages. Although DRC has recently adopted recommendations that persons with HIV and TB co-infection should be automatically placed in the third or fourth clinical stage, the adoption of this principle is recent in DRC, where CD4 count was also used to classify patients, particularly when viral load was not available. The severity of HIV in co-infected PLHIV may be attributable to TB as an opportunistic infection that is associated with the late stages of HIV infection when patients have progressed to either the moderately symptomatic or severely symptomatic stage and have reduced immune ability to fight off pathogens. To increase the quality of life and decrease the likelihood of comorbidities, such as TB, HIV programs should proactively target PLHIV because they are all considered at risk for co-infection; thus, the diagnosis could be made during the early stages of their infection, appropriate care and treatment can be provided, and steps can be taken to ensure compliance to care that is conducive to preventing or slowing progression to the later stages of infection.

Our study findings suggested that gender differences exist in the severity of HIV, with female patients more likely to be at stage 1 rather than stage 3 or 4. These differences can be attributable to gender differences in healthcare-seeking behavior due to social and cultural influences. In DRC, men show a delayed healthcare-seeking behavior, which negatively impacts the severity of their HIV stage. Existing literature on healthcare-seeking behavior has shown that gender and other characteristics do influence when a patient pursues care [16,17]. Studies have shown that women tend to have more contact with the health system than men, and the rurality status of the patient’s location affects the accessibility of available health care [18,19,20,21,22,23]. The current study is consistent with the existing literature in that our results showed significantly higher odds of being in the early stages of HIV infection in female patients than males. Additionally, the rurality status of the health zone was associated with the high-severity stage (stage 3 or 4) when compared to patients in the urban health zones. Rurality status shapes many social determinants of health. Residents in rural areas face inequities, including lower median household incomes, decreased access to postsecondary education, increased likelihood of living in poverty, risk of being uninsured, and shortage of qualified health workforce, all of which may result in poorer access to and quality of healthcare, which in turn may put residents at higher risk for stage 3 or 4 HIV progression. Remote locations of rural areas contribute to poor access to care, as rural residents must travel long distances to reach the nearest healthcare provider. Long-distance travels can amount to absenteeism from work for an initial appointment and follow-ups, resulting in non-utilization or delay in the utilization of healthcare services. It may be important to consider these socio-economic factors as determinants of health in rural areas and formulate policies and allocate resources for addressing barriers to care in these areas. Recent studies in sub-Saharan countries have shown similar barriers to access and treatment in rural outpatient clinics when compared to outpatient clinics in urban areas [24,25,26].

Duration on ART also had an impact on the stage of clinical progression of the infection among the study participants. The current study found that HIV patients who were on ART for less than 40 months were less likely to have progressed to later stages of stage 3 (moderately symptomatic) or stage 4 (severely symptomatic or AIDS) than patients who were on ART for more than 40 months. The longer duration of a patient on ART signifies that a person has been somewhat immunocompromised for a longer duration. TB is considered one of the opportunistic infections, so the probability of such infection increases, particularly as patients move into stage 3 or 4 of HIV infection, which is the AIDS stage. The higher odds of patients on more prolonged ART therapy to be at HIV stages 3 and 4 may also be due to the possible resistance to treatment (ART) or irregularity/non-compliance to ART, due to medication fatigue. Additional research may be needed to explain this discrepancy in the severity of the HIV progression stage.

The study limitations pertain to secondary sources of data and inconsistencies in viral load measurement across years in which data were collected, which impeding our ability to analyze whether the viral load was associated with the clinical stage. Additionally, the analyses were based on a de-identified, limited-variable dataset extracted from the program database with a small number of variables. Many potential socioeconomic contributors to the management of HIV were not available for these analyses. Finally, DRC has recently adopted recommendations that persons with HIV and TB co-infection should be automatically placed in the 3rd or 4th clinical stage. The inclusion of multiple years of data may confuse some readers about our results showing some persons with TB/HIV co-infection in the 1st or 2nd clinical stage. The recent adoption of the guidelines to classify all patients with TB/HIV co-infection into stage 3 or stage 4 means that the earlier use of alternative methods of classifying (e.g., based on CD4 count) may have resulted in an over-enumeration of the patients in stages 1 and 2, and under-enumeration in stages 3 and 4. Regardless of these limitations, the study findings are valuable given this study’s strengths. The first strength pertained to the selection of the topic and research collaborations. Consistent with principles of practice-relevant/practice-based research, the study topic was selected after a formal consultation with the stakeholders and implementing partners of the National HIV/AIDS Program, and critical feedback was obtained from selected stakeholders. The topic was considered of importance to the work of these and similar clinics given that understanding ways to reduce the severity of HIV symptoms reflected in WHO clinical stages is critical to reducing the burden of HIV on individuals, families, and communities. Secondly, the study sample size was robust, allowing multivariable modeling. Further understanding the comorbidities associated with severe symptomatic stages and preventing them will be two-fold advantageous. First, the burden of resources will decrease from the program perspective. Secondly, for the patients, the reduced morbidity will mean motivation for higher compliance with the treatment regimen leading to viral suppression and efficient management of the disease.

## 5. Conclusions

This study used principles of practice-based research to highlight health status disparities, indicative of health inequities inherent in social determinants of health, among HIV patients on ART in HIV clinics in DRC. The study showed health disparities in the severity of HIV progression noted by WHO stages of HIV progression by rurality status, gender, and duration on ART, among other factors. Our findings may fuel important debates on the need for the HIV programs in DRC to move away from a traditional healthcare paradigm of provision of routine clinical care without building holistic support services that cater to the special needs of special-risk subgroups. A paradigm shift from a focus on exclusively managing individual HIV status to a proactive population health focus is imperative, wherein health inequities are identified and addressed upstream in order to prevent downstream disparities in HIV care utilization, HIV care and treatment outcomes, and HIV transmission. Additionally, the severity of AIDS progression is much higher among patients with TB co-infection compared those without such co-infection, which suggests a need for customized approaches and clinical regimens for this high-risk population. Given the stigma associated with HIV, TB, and COVID-19, the need for customized care is even more critical in the COVID-19 era, due to the intersectionality of vulnerabilities from these diseases.

## Figures and Tables

**Table 1:** Descriptive demographic and clinical characteristics of patients in HIV/AIDS clinics of Haut-Katanga and Kinshasa provinces, Democratic Republic of Congo, 2014–2019.

Demographic and Clinical Characteristics	*n*	Percent
WHO Clinical Stage		
Stage 1	18,515	41.9
Stage 2	10,116	22.9
Stage 3	13,565	30.7
Stage 4	1960	4.4
Patient Sex		
Female	34,134	69.0
Male	15,326	31.0
Age at the time of the first visit		
Younger than 15 years	4769	10.3
15 years or older	41,454	89.7
TB status of patient		
No TB	43,218	96.4
TB present	1631	3.6
Province		
Haut-Katanga	14,596	29.5
Kinshasa	34,864	70.5
Rurality/Urbanicity of the Health Zone		
Rural	2790	5.6
Semi-rural	5780	11.7
Urban	40,890	82.7
Duration on ART (months)		
Less than 3.23 months	11,566	25.0
3.23 to 14.52 months	11,540	25.0
14.53 to 40.37 months	11,549	25.0
More than 40.37 months	11,542	25.0

Note: *n*, the total number of patients in the study, was 49,460. Abbreviations: *n*, number of patients; TB, tuberculosis; ART, antiretroviral therapy.

**Table 2:** Bivariate association between demographic/clinical characteristics and WHO clinical stage among patients in HIV/AIDS clinics of Haut-Katanga and Kinshasa provinces, Democratic Republic of Congo, 2014–2019.

Demographic and Clinical Characteristics	WHO Clinical Stage			
	Stage 1	Stage 2	Stage 3 or 4	*p*-Value
	%	%	%	
TB status of patient				<0.001
No TB	43.00%	23.00%	34.00%	
TB present	17.80%	14.30%	67.90%	
Patient sex				<0.001
Female	43.90%	22.50%	33.70%	
Male	37.60%	23.90%	38.40%	
Age at the time of the first visit				<0.001
Younger than 15 years	39.00%	25.40%	35.50%	
15 years or older	41.80%	22.60%	35.60%	
Province				<0.001
Haut-Katanga	47.60%	25.00%	27.40%	
Kinshasa	39.40%	22.00%	38.60%	
Rurality/Urbanicity of the Health Zone				<0.001
Rural	34.40%	23.30%	42.30%	
Semi-rural	38.20%	25.20%	36.60%	
Urban	43.10%	22.50%	34.40%	
Duration on ART (months)				<0.001
Less than 3.23 months	45.30%	24.10%	30.60%	
3.23 to 14.52 months	48.30%	21.70%	30.00%	
14.53 to 40.37 months	41.80%	23.00%	35.20%	
More than 40.37 months	30.30%	22.70%	47.00%	

Note: *n*, the total number of patients in the study was 49,460. Abbreviations: TB, tuberculosis; ART, antiretroviral therapy.

**Table 3:** Multinomial logistic regression of WHO clinical stage among patients in HIV/AIDS clinics of Haut-Katanga and Kinshasa provinces, Democratic Republic of Congo, 2014–2019.

Demographic and Clinical Characteristics	WHO Clinical Stages							
	Stage 1 vs. Stage 3 or 4				Stage 2 vs. Stage 3 or 4			
	AOR	95% C.I. for		*p*	AOR	95% C.I. for		*p*
		LL	UL			LL	UL	
TB status of patient								
No TB	5.73	4.98	6.59	<0.001	3.6	3.1	4.2	<0.001
TB present *								
Patient sex								
Female	1.35	1.29	1.42	<0.001	1.09	1.03	1.15	0.003
Male *								
Age at the time of the first visit								
Younger than 15 years	1.01	0.94	1.09	0.734	1.14	1.05	1.24	0.003
15 years or older *								
Province								
Haut-Katanga	2.25	2.12	2.38	<0.001	1.85	1.73	1.98	<0.001
Kinshasa *								
Rurality/Urbanicity of the Health Zone								
Rural	0.32	0.29	0.36	<0.001	0.5	0.44	0.56	<0.001
Semi-rural	0.79	0.74	0.85	<0.001	1	0.92	1.08	0.98
Urban *								
Duration on ART (months)								
Less than 3.23 months	2.47	2.31	2.64	<0.001	1.74	1.61	1.87	<0.001
3.23 to 14.52 months	2.6	2.43	2.77	<0.001	1.54	1.43	1.66	<0.001
14.53 to 40.37 months	1.77	1.65	1.88	<0.001	1.28	1.19	1.38	<0.001
More than 40.37 months *								

Abbreviations: AOR, adjusted odds ratio; CI, confidence interval; LL, lower limit; UL, upper limit; TB, tuberculosis; ART, antiretroviral therapy. Note: the number of patients included in multivariate analysis was 41,250. Bold indicates statistically significant at *p* < 0.05; “*” indicates the reference category.

## Data Availability

The program implementing partners required that data be destroyed after publication. Original data are archived with the original data owners mentioned in the methods section.

## References

[1] WHO (2017). Global Health Observatory Data Repository.

[2] Fauci A.S. (2005). Human immunodeficiency virus disease: AIDS and related disorders. Harrison’s Principles of Internal Medicine.

[3] Miedema F. (2006). T cell dynamics and protective immunity in HIV infection: A brief history of ideas. Curr. Opin. HIV AIDS.

[4] Murray P.R., Rosenthal K.S., Pfaller M.A. (2020). Medical Microbiology.

[5] Pantaleo G., Fauci A. (1996). Immunopathogenesis of HIV infection. Annu. Rev. Microbiol..

[6] WHO (2005). Interim WHO Clinical Staging of HIV/AIDS and HIV/AIDS Case Definitions for Surveillance: African Region.

[7] Weinberg J.L., Kovarik C.L. (2010). The WHO clinical staging system for HIV/AIDS. AMA J. Ethics.

[8] Malamba S.S., Morgan D., Clayton T., Mayanja B., Okongo M., Whitworth J. (1999). The prognostic value of the World Health Organisation staging system for HIV infection and disease in rural Uganda. AIDS.

[9] Langford S.E., Ananworanich J., Cooper D.A. (2007). Predictors of Disease Progression in HIV Infection: A Review. AIDS Res. Ther..

[10] Charles M., Boyle B. (2002). Excess and access: The continuing controversy regarding HIV and health care in Africa. AIDS Read..

[11] UNAIDS (2015). Understanding Fast Track: Accelerating Action to End the AIDS Epidemic by 2030. UNAIDS Geneva..

[12] UNAIDS (2019). Country Fact Sheet: Democratic Republic of the Congo. https://www.unaids.org/en/regionscountries/countries/democraticrepublicofthecongo.

[13] Benzekri N.A., Sambou J.F., Ndong S., Tamba I.T., Faye D., Diallo M.B., Diatta J.P., Faye K., Sall I., Sall F. (2019). Prevalence, Predictors, and Management of Advanced HIV Disease among Individuals Initiating ART in Senegal, West Africa. BMC Infect. Dis..

[14] Avert Global Information and Education on HIV and AIDS: The HIV Treatment Cascade. https://www.avert.org/professionals/hiv-programming/treatment/cascade.

[15] (2017). IBM SPSS Statistics for Windows, Version 25.0.

[16] Langeni T. (2007). Contextual factors associated with treatment-seeking and higher-risk sexual behaviour in Botswana among men with symptoms of sexually transmitted infections. Afr. J. AIDS. Res..

[17] Tsadik M., Lam L., Hadush Z. (2019). Delayed health care seeking is high among patients presenting with sexually transmitted infections in HIV hotspot areas, Gambella town, Ethiopia. HIV AIDS.

[18] Thompson A., Anisimowicz Y., Miedema B., Hogg W., Wodchis W., Aubrey-Bassler K. (2016). The influence of gender and other patient characteristics on health care-seeking behaviour: A QUALICOPC study. BMC Fam. Pract..

[19] Das M., Angeli F., Krumeich A., van Schayck O. (2018). The gendered experience with respect to health-seeking behaviour in an urban slum of Kolkata, India. Int. J. Equity Health.

[20] Lim M.T., Lim Y.M.F., Tong S.F., Sivasampu S. (2019). Age, sex and primary care setting differences in patients’ perception of community healthcare seeking behaviour towards health services. PLoS ONE.

[21] Wang Y., Hunt K., Nazareth I., Freemantle N., Petersen I. (2013). Do men consult less than women? An analysis of routinely collected UK general practice data. BMJ Open.

[22] Ashour M. (2017). Individual and socioeconomic disparities in health-seeking behaviours and out-of-pocket payments in the Gaza Strip in 2013: Results from a household survey. Lancet.

[23] Rathore M.A., Rashid Z., Mashhadi S.F., Rathore M.A., Sharif I. (2020). Health Care Seeking Behavior among Newly Diagnosed Human Immunodeficiency Virus Cases in Rawalpindi. Pak. Armed Forces Med. J..

[24] Fokam J., Nangmo A., Wandum C., Takou D., Santoro M.M., Nlend A.N., Ateba F.N., Ndombo P.K., Kamgaing N., Kamta C. (2020). Programme quality indicators of HIV drug resistance among adolescents in urban versus rural settings of the centre region of Cameroon. AIDS Res. Ther..

[25] Zegeye E.A., Mbonigaba J., Kaye S., Johns B. (2019). Assessing the cost of providing a prevention of mother-to-child transmission of HIV/AIDS service in Ethiopia: Urban-rural health facilities setting. BMC Health Serv. Res..

[26] Mutembo S., Mutanga J.N., Musokotwane K., Kanene C., Dobbin K., Yao X., Li C., Marconi V.C., Whalen C.C. (2019). Urban-rural disparities in treatment outcomes among recurrent TB cases in Southern Province, Zambia. BMC Infect. Dis..

